# CIEGAN: A Deep Learning Tool for Cell Image Enhancement

**DOI:** 10.3389/fgene.2022.913372

**Published:** 2022-07-04

**Authors:** Qiushi Sun, Xiaochun Yang, Jingtao Guo, Yang Zhao, Yi Liu

**Affiliations:** ^1^ Beijing Key Lab of Traffic Data Analysis and Mining, School of Computer and Information Technology, Beijing Jiaotong University, Beijing, China; ^2^ State Key Laboratory of Natural and Biomimetic Drugs, MOE Key Laboratory of Cell Proliferation and Differentiation, Beijing Key Laboratory of Cardiometabolic Molecular Medicine, Institute of Molecular Medicine, College of Future Technology, Peking University, Beijing, China

**Keywords:** cell image, image enhancement, long-term imaging, deep learning, generative adversarial network

## Abstract

Long-term live-cell imaging technology has emerged in the study of cell culture and development, and it is expected to elucidate the differentiation or reprogramming morphology of cells and the dynamic process of interaction between cells. There are some advantages to this technique: it is noninvasive, high-throughput, low-cost, and it can help researchers explore phenomena that are otherwise difficult to observe. Many challenges arise in the real-time process, for example, low-quality micrographs are often obtained due to unavoidable human factors or technical factors in the long-term experimental period. Moreover, some core dynamics in the developmental process are rare and fleeting in imaging observation and difficult to recapture again. Therefore, this study proposes a deep learning method for microscope cell image enhancement to reconstruct sharp images. We combine generative adversarial nets and various loss functions to make blurry images sharp again, which is much more convenient for researchers to carry out further analysis. This technology can not only make up the blurry images of critical moments of the development process through image enhancement but also allows long-term live-cell imaging to find a balance between imaging speed and image quality. Furthermore, the scalability of this technology makes the methods perform well in fluorescence image enhancement. Finally, the method is tested in long-term live-cell imaging of human-induced pluripotent stem cell-derived cardiomyocyte differentiation experiments, and it can greatly improve the image space resolution ratio.

## Introduction

Microscopic imaging and fluorescence imaging technology have brought great convenience to biological research, and allow researchers to visually observe subcellular structures and the interaction between cells. The emergence of long-term live-cell imaging technology has made it possible to observe the cultivation and growth process of cells, which is expected to explain more biological phenomena over time. In particular, the dynamics of changes in cellular and subcellular structures and protein subcellular localization, and the dynamic process of cell differentiation and reprogramming were studied. It is crucial to decipher the mechanism behind the dynamic heterogeneous cellular responses.

Many studies require long-term imaging of living cells, so brightfield imaging should be carried out for further analysis to keep cells alive. The brightfield imaging process is simple without a fluorescent staining operation and the noise introduced into the experimental system is quite low. The non-intrusive experimental method shows great advantages: 1) no complex experimental operations, 2) does not introduce noise into the experimental system, and 3) does not interfere and destroy the cells themselves, while the phototoxicity can be reduced to a minimum. Since long-term live-cell imaging has such advantages, there have been many studies.

Smith et al. (2010) used high-resolution time-lapse imaging to track the reprogramming process from single mouse embryonic fibroblasts (MEFs) to induced pluripotent stem (iPS) cell colonies over 2 weeks. Schroeder (2011) conducted continuous long-term single-cell tracking observations of mammalian stem cells and found a set of technical solutions for long-term imaging and tracking. McQuate et al. (2017) established a pipeline for long-term live-cell imaging of infected cells and subsequent image analysis methods for *Salmonella* effector proteins SseG and SteA. Chen et al. (2010) developed a machine learning-based classification, segmentation, and statistical modeling system based on a time-lapse brightfield imaging analysis system to guide iPSC colony selection, counting, and classification automatically. In their research, AlexNet and hidden Markov model (HMM) technology were used. Buggenthin et al. (2017) used long-term time-lapse microscopy data and single-cell tracking annotation to prospectively predict differentiation outcomes in differentiating primary hematopoietic progenitors. They propose a convolutional neural network (CNN) combined with a recurrent neural network (RNN) architecture to process images from brightfield microscopy and cell motion. They predicted primary murine hematopoietic stem and progenitor cells (HSPCs) differentiating into either the granulocytic/monocytic (GM) or the megakaryocytic/erythroid (MegE) lineage. Wang et al. (2020) developed a live-cell imaging platform that tracks cell state changes by incorporating endogenous fluorescent labels. It can minimize the perturbation to cell physiology when processing live-cell imaging. In the field of cell differentiation and reprogramming, continuous long-term single-cell observation provides an insight into the mechanisms of cell fate. Even in the field of education, the low-cost long-term live-cell imaging platform also has high application prospects (Walzik et al., 2015).

**FIGURE 1:**
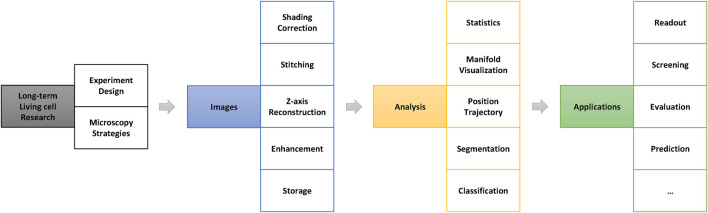
General processing pipeline for long-term live-cell research. The image preprocessing stage (blue part in this figure) is very important in the entire research pipeline and directly determines the accuracy and results of the further analysis.

A summary of the general processing pipeline of long-term live-cell imaging research is shown in Figure 1. Once the research question has been set up, appropriate microscopy strategies must be tailored according to the experimental system to be used at the beginning of the study. In addition, it is necessary to balance the trade-offs between the image space resolution ratio, experimental throughput, and imaging speed (Weigert et al., 2018), limited by imaging technology and cost.

**FIGURE 2:**
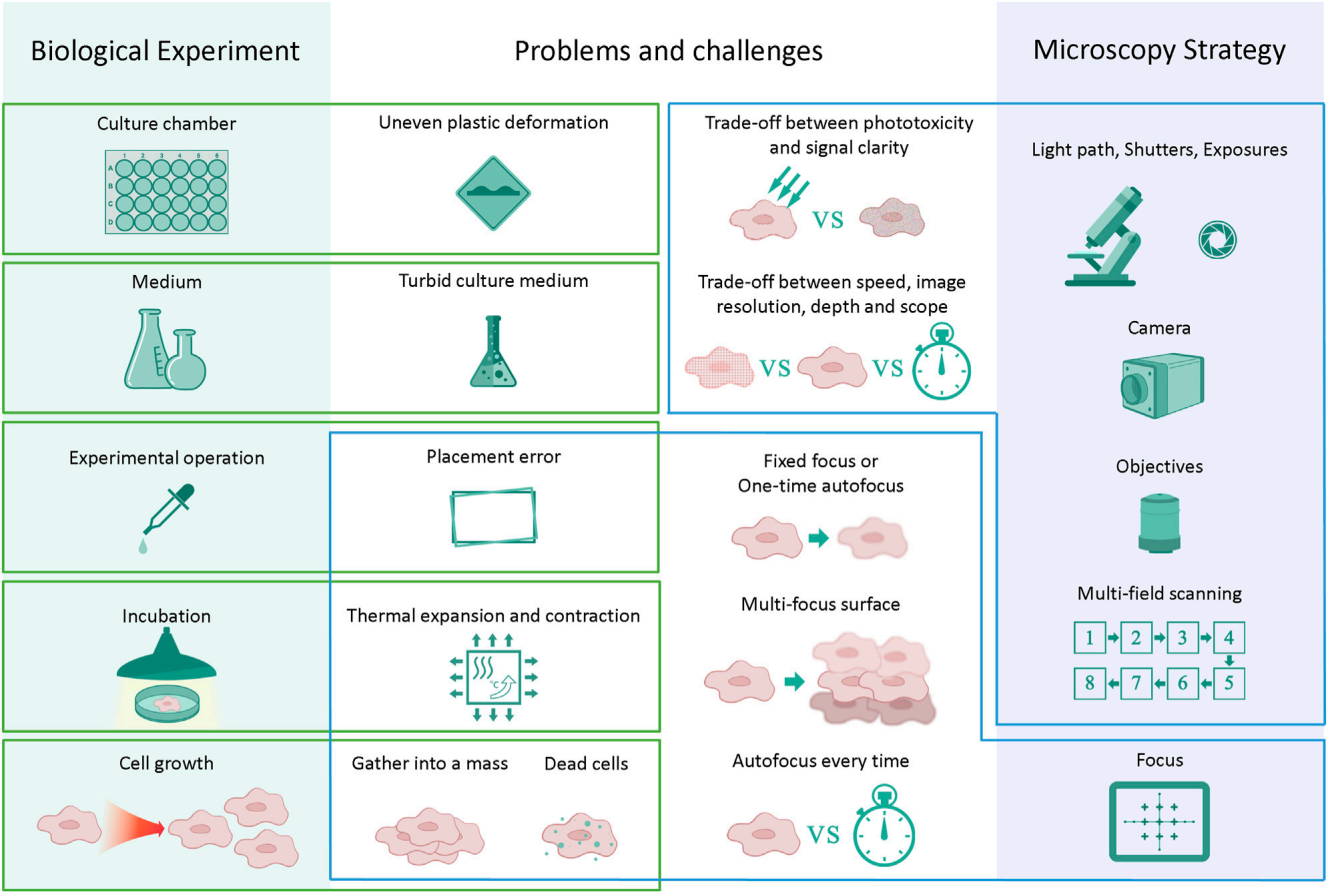
Challenges in long-term live-cell imaging. Changes in individual steps or components may influence the next series of steps and cause a reduction in image quality. Trade-offs must be made between time and image quality in almost every long-term experiment.

However, many difficulties and challenges arise in actual long-term imaging experiments. This is the congenital deficiency of long-term live-cell imaging. It takes considerable effort to maintain regular cell culture conditions while performing long-term high-resolution imaging (Skylaki et al., 2016). For example, the obtained photos may not be as sharp and distinguishable as traditional fluorescent label imaging because the noninvasive label-free observation method has no conspicuous calibrations. At the same time, it is necessary to reduce the phototoxicity to a range that can be tolerated in an experimental system while exposing the live cells to the transmitted light in long-term incubation. Thus, it reduces the signal-to-noise ratio of image acquisition because the light intensity is limited. Moreover, a large number of cells will aggregate into clusters as a result of cell growth and culture in long-term live-cell culturing. The sudden growth of cells in a mass can cause loss of the focal surface. Dead cells will become pollutants, float up and block the view. Furthermore, artificial placement errors will be introduced in the long-term experimental system. For example, the medium was changed every certain period of time to maintain regular cell survival or differentiation. Nevertheless, thermal expansion and contraction of the culture chamber are caused by temperature changes during the movement of the culture chamber in and out of the thermostatic incubator. The quality and clarity of the medium will lead to a decrease in the quality of the acquired images. Some of the conditions that cause blurring in long-term live-cell imaging are shown in Figure 2. Most importantly, it does not leave enough time for the researcher to take another image because many cellular dynamic response processes are rare and occur quickly. On the other hand, it always takes several days or weeks to reproduce the entire biological experiment again, which wastes considerable time. Therefore, the industry urgently needs a tool that can efficiently improve the quality of once-taken bad images and reconstruct high-quality microscopic images of cells.

Image processing methods such as image inpainting or image completion can be used to restore imperfect cell images. The rapid progress of deep learning technology (Hinton et al., 2006; Hinton and Salakhutdinov, 2006) and deep convolutional neural networks (CNNs) has led to many new applications in computer vision and image processes. The emergence of generative adversarial networks (GANs) (Goodfellow et al., 2014) has brought almost a leap in image generation, inpainting, repair, and completion. A conditional generative adversarial net (CGAN) (Mirza and Osindero, 2014) can generate custom outputs by adding class information to the model. The best of these methods in image processing is deep convolutional generative adversarial networks (DCGANs) proposed by Radford et al. (2016), which replace fully connected layers in the original GANs with the convolutional layers in both the generator and the discriminator. Recently, many excellent image repair methods based on DCGAN structures have been proposed for real-world photo restoration, such as those by Pathak et al. (2016), Iizuka et al. (2017), and Yu et al. (2018). These methods work very well on landscape, architecture, or portrait retouching.

Recently, image-to-image translation tasks have been proposed to address image style transfer, which aims to translate an input image from a source domain to a target domain. The “pix2pix” proposed by Isola et al. (2017) is an image translation method based on conditional adversarial networks, which has shown the super ability of street scene restoration in the real world. “Pix2pix” uses input–output image pairs as training data, and pixel-wise reconstruction loss coupled with adversarial loss is used to optimize the model building process.

On the other hand, the single-image super-resolution (SISR) method has emerged to recover a high-resolution (HR) image from a single low-resolution (LR) image. Wei et al. proposed the “artificial neural network accelerated-photoactivated localization microscopy” (ANNA-PALM) method for reconstructing high-quality cell super-resolution views from sparse, rapidly acquired, single-molecule localization data and widefield images (Ouyang et al., 2018). Based on the “pix2pix” architecture, this method greatly facilitates studies of rare events, cellular heterogeneity, or stochastic structures. The super-resolution generative adversarial network (SRGAN) proposed by Ledig et al. (2017) is one of the milestones in single image super-resolution, and it significantly improves the overall visual quality of reconstruction over traditional methods. The SRGAN innovatively uses content loss coupled with adversarial loss instead of PSNR-oriented loss as the objective function. There are many variants of SRGAN methods, such as the enhanced SRGAN (ESRGAN) proposed by Wang et al. (2018) and the practical restoration application ESRGAN (Real-ESRGAN) proposed by Wang et al. (2021b). In an ESRGAN, a residual-in-residual dense block (RRDB) was introduced to the model as the basic network building unit, which combines a multilevel residual network and dense connections. The RRDB can further improve the recovered textures by adopting a deeper and more complex structure than the original residual block in the SRGAN. The Real-ESRGAN uses the U-net discriminator with spectral normalization as a modification to the ESRGAN to increase the discriminator capability and stabilize the training dynamics. Therefore, it is better at restoring most real-world images than previous works, especially low-quality web images or videos with compression degradations.

While the aforementioned methods perform well on macroscopic photographs such as street views, these methods do not perform well enough in the reconstruction of biological images, which require very precise fine structure recovery. Therefore, inspired by the methods in the field of image completion and image super-resolution (Wang et al., 2018, 2021a, 2021b; Rad et al., 2019; Qiao et al., 2021), we propose a cell image-enhanced generative adversarial network (referred to as CIEGAN) for image enhancement to address the challenges mentioned previously. In addition to using adversarial loss, the CIEGAN introduced perceptual losses comprising feature reconstruction loss and style reconstruction loss (Gatys et al., 2015; Johnson et al., 2016), which greatly improves the image restoration efficiency of the model. Coupled with image reconstruction loss and the total variation regulator, our method can solve various blurry problems of biological cell images. This method is very convenient and especially optimized for long-term live-cell imaging. Moreover, it can increase the imaging speed because there is no need to take more Z-axes layers for focus finding. Researchers can have more time to scan more conditions or increase the frequency of image acquisition. Furthermore, it can handle the force majeure during cell culture: cell clumping, cell bulging or blurring caused by floating dead cells, etc., even if the blur was caused by the beating of the differentiated mature cardiomyocytes. Nevertheless, the processing is fast, of low cost, and can easily be extended to other photos. It is convenient for researchers to obtain the differentiation or development trajectories of cell lines from the image stream and conduct research such as differentiation trajectory tracking, subtype search, or protein subcellular localizations (Aggarwal et al., 2021).

We applied the CIEGAN to long-term live-cell imaging of a human-induced pluripotent stem cell (hiPSC)-derived cardiomyocyte (hiPSC-CM) differentiation system, which greatly enhanced the quality of brightfield cell images. Through the comparison of results, it is found that the CIEGAN based on generative adversarial networks is better than the traditional image enhancement algorithm and can use the original blurred images to reconstruct sharper images. The information entropy of the enhanced image is increased and its resolution ratio is also significantly improved. At the same time, we also found that it is quite suitable for the enhancement of fluorescence images.

## Materials and Methods

This section describes the experimental steps and methods of the hiPSC-CM differentiation system. In addition, microscopy techniques and strategies have been used in the image data acquisition of live cells in a long-term culture. Notably, there are many challenges in the acquisition of microscopic images in long-term live-cell culture systems, and in some cases, image quality is sacrificed to balance the pros and cons. Here, the main technology and workflow of a cell image enhancement GAN are shown in detail and explained how a CIEGAN improves the sharpness of microscopic cell images. Finally, the deployment and training process of the model are also described.

### Human-Induced Pluripotent Stem Cell Culture and Differentiation

**FIGURE 3:**
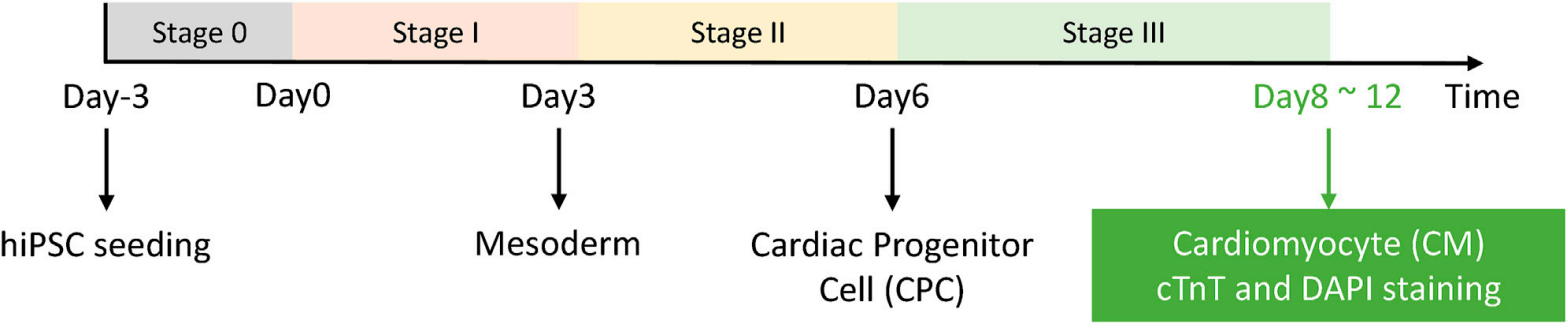
HiPSC-CM experimental system. Stage 0 is the hiPSC seeding and growth stage, and the differentiation process starts from day 0. After stage III, the cells were fixed and stained for readout and further analysis.

Our experimental system is the differentiation induction process of human pluripotent stem cells into cardiomyocytes. The main differentiation process is shown in Figure 3, and images were captured and saved throughout the process.

First, the iPSC-18 cell line was chosen for induction experiments. iPSC-18 cells (Y00300, Takara) were routinely cultured in a PGM1 medium (CELLAPY) on growth factor–reduced Matrigel (corning)-coated 6-well plates. iPSC-18 cells were passaged every 4 days using EDTA (Gibco). hiPSCs were split into a CDM medium (Cauliscell Inc.) at a ratio of 1:10 before differentiation in 24-well or 96-well plates. When they attained ∼80–90% confluence, the medium was changed to a RPMI 1640 medium (RPMI, Gibco), 1x B27 without insulin (Gibco), and 100 U penicillin (Gibco), or RPMI+B27 minus, for shorting. During the first 48 h, hiPSCs were treated with CHIR99021 (CHIR, a WNT activator). From 48 to 72 h (day 3), the medium was changed to RPMI+B27 minus. During days 4–5, RPMI+B27 minus medium was supplemented with IWR1 (a WNT inhibitor). On day 6, IWR1 was withdrawn instead of the RPMI+B27 minus medium. After day 7 through to the end of the differentiation process (up to 14 days), the RPMI 1640 medium (RPMI, Gibco), 1 x B27 (Gibco), and 100 U penicillin (Gibco) were used and refreshed every 3 days.

### Immunofluorescence Staining

After the final stage of induction (stage III: day 8–day 12), the cells were fixed in 4% paraformaldehyde (DING GUO) for 20 min at room temperature, permeabilized, and blocked in 3% normal donkey serum (Jackson) and 0.1% Triton X-100 for 45 min at room temperature. Then, the cells were incubated with a cTnT antibody (Thermo, MA5-12960, use 1:300) overnight at 4°C in PBS plus 0.1% Triton X-100 and 3% normal donkey serum (Jackson). The cells were washed with PBS and then incubated with secondary antibodies for 1 h at 37°C in a dark environment in PBS and 1% bovine serum albumin (BSA). Nuclei were stained with Hoechst 33342 (Yeasen) for 5 min at room temperature.

### Microscopic Image Acquisition

The irresistible degradation of image quality introduced in long-term live-cell experiments has been described previously. Errors are introduced during the culture medium changing operation on average every 24–48 h according to the experimental steps of the iPSC-CM differentiation system. An elaborate microscopy strategy must be carefully designed to reduce these errors as much as possible. A good microscopy strategy can maximize the image quality and speed up photographing so that higher throughput experimental image data can be obtained within one culture cycle. It is essential to find a balance between the imaging resolution ratio, experimental throughput, and imaging speed (Figure 2).

Because the imaging field of view of the microscope is limited to the optical device itself and light path design, multiple scanning imaging is required to expand the field of observation view, and the whole picture is stitched after the image acquisition. Therefore, the larger the observation field to be photographed, the more time-consuming it will be. It will cost more time to scan more culture chambers in a parallel multi-condition comparison observation of cell differentiation or reprogramming studies. On the other hand, it will further reduce the imaging speed of the system to perform the Z-axis layer imaging if the three-dimensional structure needs to be observed. Therefore, it is necessary to accelerate the imaging speed of each imaging experimental cycle to ensure the acceptable frequency of observation. If a higher imaging speed is required, a narrower imaging breadth is to be obtained, and vice versa. You cannot have your cake and eat it, too.

In addition, there are many options for different focusing strategies in an experiment. If the fixed focus or the one-time autofocus has been chosen, the out-of-focus caused by various emergencies in the long-term live-cell imaging process cannot be handled. For example, culture chamber expansion and contraction are caused by temperature or dead cell contamination. In particular, cells are raised into multiple layers because of cell growth. At this time, each layer of cells has its own focus surface due to the overlapping of multiple cell layers. Only multiple Z-axis microscopic imaging can obtain each sharp view of the overlapping cells. Notably, different cell types have different clonal heights, and the optimal focus surface may span more than 50–60 μM or even 100 μM in our iPSC-CM differentiation system.

On the other hand, if the autofocus every-time mode has been chosen, it is almost impossible to carry on the experiment because the focusing process will take plenty of time. Each well requires a 5 × 5 pattern mosaic tile stitching to obtain a square field of view of approximately 6.3 mm*6.3 mm according to the 96-well plates in this research. If only the center of each well is used as the focus reference point, then it will take approximately 48 min for 96 focus points to perform high-speed hardware autofocus. Moreover, if autofocus needs to be performed on each tile, the total time spent focusing will be 25 times larger, which is overtime to an incredible 20 h. Unfortunately, more focal points per well are required for the 24-well plates because of the larger culture area of each well. There is a way to combine a one-time autofocus strategy and multiple Z-axis imaging, then select the sharpest layer for use after imaging experiments (more images are shown in Supplementary Materials), but it also comes at the expense of time. These limits necessitate trade-offs between the imaging resolution ratio, experimental throughput, and imaging speed (Figure 2).

Here, the “Celldiscoverer 7,” a long-term live-cell culture instrument manufactured by Carl Zeiss, is used. It has an internal incubator to ensure regular cell growth, and the cell culture environment is kept stable at 37 °C with 5% CO_2_. The ORCA-Flash 4.0 V3 digital CMOS camera is used as HD picture acquisition equipment. The effective resolution of the camera is 2,048*2,048 pixels. The objective is a ZEISS Plan-Apochromat ×5 objective. The objective can easily handle thin and thick vessel bottoms made of glass or plastic, which is essential to the hiPSC-CM differentiation system because our cells can only grow on plastic. With a ×2 tube lens, it achieves 10x/0.35 magnification and spatial resolution. Finally, the resolution ratio of all the photos is 0.65 μM per pixel.

For culture chambers, 96-well and 24-well plates produced by Falcon are used. A 2,048*2,048-pixel photo can cover a square of approximately 1.33*1.33 mm because of the resolution ratio of 0.65 μM per pixel. Therefore, the scanning imaging method was adopted for image acquisition, and the whole images were stitched after the imaging experiments. The larger the observation field is, the more mosaic tiles will be needed.

Multiple Z-axis layers are photographed to study the multiple Z-axis layer aggregation of the cells in the iPSC-CM differentiation process and to find the cause of blurring, and more importantly, to obtain the training data for the model. Eleven, seven, and five layers at 1.5 μM, 6 μM, and 18 μm intervals with total vertical distances of 15 μM, 36 μM, and 72 μM, respectively, were obtained for study (the images are shown in the Supplementary Materials).

Finally, the microscopic images were acquired by Carl Zeiss ZEN software version V2.5, and the images were saved in the CZI format or PNG format. A real-time microscopic image processing framework has been compiled for the long-term live-cell imaging system. It can automatically acquire and perform image preprocessing correspondingly, including image stitching and image segmentation. The segmented images will be sent to the CIEGAN for image enhancement for further analysis.

### Cell Image Enhancement Generative Adversarial Networks

The deep convolutional generative adversarial network structure (Goodfellow et al., 2014; Radford et al., 2016) is adopted as the main body of the model to reconstruct high-quality and high-resolution images from low-quality microscopic cell images.

The GAN architecture in our model comprises a pair of generators and discriminators. Typically, the generator is trained to generate fake samples from random noise vector *z*. However, in our model, we take the blurred original image as the input *z* to enhance it. The input image flows through a pair of our carefully designed encoder-decoder-like structures in the generator. The latent implicit representation of the input image is obtained from the encoder module. The output image is precisely reconstructed using the information provided by the latent representation. On the other hand, the discriminator is trained to distinguish between the real cell images and the generated fake images. This framework can be represented as a two-player min-max game between generator G and discriminator D with value function V(D,G) :

$$\underset{G}{\min}\underset{D}{\max}V\left(D,G\right)={Ε}_{x∼p_{data}\left(x\right)}\left[\log\left(D\left(x\right)\right)\right]+{Ε}_{z∼p_{z}\left(z\right)}\left[\log\left(1-D\left(G\left(z\right)\right)\right)\right].$$ (1)

In Eq. 1, *x* represents the real-world high-resolution cell image examples. Discriminator D was trained to maximize the probability of assigning the correct label to both generated enhanced samples from G and the real-world cell image examples. At the same time, the generator G was trained to minimize log(1−D(G(z))) simultaneously, that is, let the generated fake samples deceive the discriminator D to the maximum extent.

In the GAN structure, only the strongest generator survives in the game, which is very suitable for image restoration tasks. The adversarial loss ensures a high degree of realism of the image, making the image more natural and realistic. The following description will use $\hat{x}$ to represent $\hat{x}=G\left(z\right)$ , the generated image samples for brevity. The adversarial loss of the discriminator is formulated as Eq. 2:

$$L_{adv_{D}}=\log\left(D\left(x\right)\right)+\log\left(1-D\left(\hat{x}\right)\right).$$ (2)

The two parts are the true labels for the ground truth samples and the false labels for the generated samples. The optimization objective of the adversarial loss of the discriminator is formulated as Eq. 3:

$$\underset{D}{\max}L_{adv_{D}}={Ε}_{x∼p_{data}}\left[\log\left(D\left(x\right)\right)+\log\left(1-D\left(\hat{x}\right)\right)\right].$$ (3)

Similarly, the adversarial loss of the generator and its optimization objective are formulated as Eqs. 4, 5:

$$L_{adv_{G}}=\log\left(D\left(\hat{x}\right)\right),$$ (4)

$$\underset{G}{\min}L_{adv_{G}}={Ε}_{x∼p_{data}}\left[\log\left(D\left(\hat{x}\right)\right)\right].$$ (5)

In the GAN structure, the latent representation can capture valuable information in the input images, and the rest of the details and textures are handed over to network parameters for completion and reconstruction. However, it is not enough to determine the network parameters precisely in the generator only by the adversarial loss of the GAN, and more penalties are required to generate more accurate images and perform more refined image restoration.

Inspired by image style transfer (Isola et al., 2017), single-image super-resolution (SISR) methods (Yang et al., 2019; Ooi and Ibrahim, 2021), and high photorealistic image synthesis (Wang et al., 2018; 2021b), a series of image reconstruction losses are introduced to the model, such as pixel-wise loss and perceptual loss.

Specifically, using only reconstruction loss can reconstruct sharp images, but the generalization abilities are poor because of its pixel-wise properties. Therefore, images generated by reconstruction loss only may have excellent results superficially but suffer overfitting problems: just a single pixel translation may lead to model failure. Therefore, combining with the perceptual loss is a wise choice. The perceptual loss enables the contents and styles of the image reappearance. The reconstruction loss, also known as pixel-wise loss, is denoted as Eq. 6:

$$L_{rec}=\frac{1}{BCHW}{\left\|x-\hat{x}\right\|}_{2}^{2}.$$ (6)

In Eq. 6, B, C, H, and W represent the training batch size, the number of channels of the image or feature map, and the height and width of the feature map, respectively.

The perceptual loss comprises two parts: the feature loss part and the style loss part. The feature perceptual loss is formulated as Eq. 7 (Gatys et al., 2015; Johnson et al., 2016):

$$L_{feat}=\sum_{i=1}^{N}\frac{1}{BCHW}{\left\|\Phi_{i}\left(x\right)-\Phi_{i}\left(\hat{x}\right)\right\|}_{2}^{2}.$$ (7)

In Eq. 7, $\Phi_{i}$ is the *i*-th layer of a pre-trained VGG-16 or VGG-19 network (Simonyan and Zisserman, 2015), and $\Phi_{i}\left(x\right)$ is the feature map of input image *x*. In the actual data flow, the shape of the feature map is the same as mentioned previously: B×C×H×W . *N* is the total number of VGG network layers. Here, we use the VGG-19 network, which is pre-trained on the ImageNet dataset (Deng et al., 2009). The style perceptual loss is formulated as Eq. 8:

$$L_{style}=\sum_{i=1}^{N}\frac{1}{BCHW}{\left\|Gram_{i}\left(x\right)-Garm_{i}\left(\hat{x}\right)\right\|}_{F}^{2}.$$ (8)

In Eq. 8, the Gram matrix can be calculated using the following formula: $Gram=AA^{T}$ , where *A* represents a matrix. Here, the use of the squared Frobenius norm instead of the squared Euclidean distance was used before.

Nevertheless, a total variation regularization is imported to the model to remove noise and mosaics from images and further reduce the spikey artifacts of the generated images. The total variation regulator is formulated as Eq. 9:

$$L_{tv}=\sum_{i,j}^{H,W}\frac{1}{BCHW}\left({\left\|{\hat{x}}_{i+1,j}-{\hat{x}}_{ij}\right\|}_{2}^{2}+{\left\|{\hat{x}}_{i,j+1}-{\hat{x}}_{ij}\right\|}_{2}^{2}\right).$$ (9)

In Eq. 9, ${\hat{x}}_{ij}$ represents a pixel from the generated enhanced image. Here, the rows and columns are calculated separately by the difference between adjacent pixels.

Finally, the loss of the CIEGAN is divided into two parts: the discriminator loss $L_{D}$ and the generator loss $L_{G}$ , shown as Eqs. 10, 11, respectively:

$$L_{D}=L_{adv_{D}},$$ (10)

$$L_{G}=\lambda_{rec}L_{rec}+\lambda_{feat}L_{feat}+\lambda_{style}L_{style}+\lambda_{tv}L_{tv}+\lambda_{adv_{G}}L_{adv_{G}}.$$ (11)

The corresponding coefficients λ are added in front of different losses in the generator loss $L_{G}$ to balance the weights of different losses.

**FIGURE 4:**
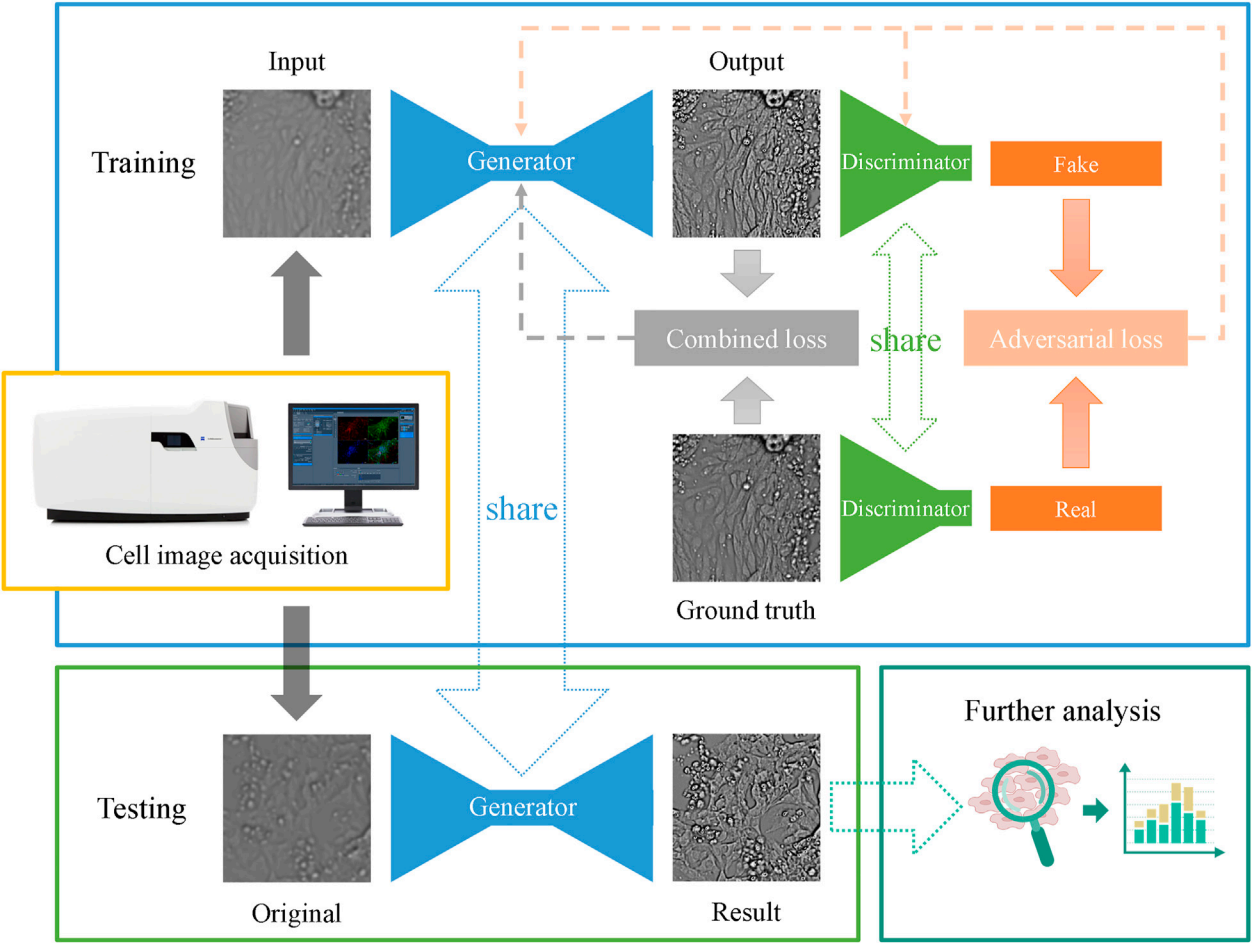
Network architecture and the main pipeline of the CIEGAN. The training process of the model is shown in the blue box, and the testing process of the model is shown in the green box in the lower-left corner, where the generator is shared. Once the model is trained, it can predict a sharp image from the original imperfect cell image.

These losses and regularizations are merged together to reconstruct high-quality images and are referred to as the combined loss shown in Figure 4. The main structure of the CIEGAN model and the training and testing processes are depicted.

### Model Building and Training

CIEGAN model coding mainly uses the TensorFlow deep learning framework (Abadi et al., 2016) and TF-slim library to build our generative adversarial nets. A cloud computing environment is used for model training and testing. The main hardware configuration list is an Intel Xeon Cascade Lake (2.5 GHz) 8-core processor, 32 GB of memory, and the CUDA computational acceleration unit is an NVIDIA T4 (with 15 GB of video memory).

Then, 128*128-pixel images, 256*256-pixel images, and 512*512-pixel images are successively tested on the CIEGAN model. According to the memory size of the CUDA unit, the network was trained using a batch size of 32, 20, and 4 images for the 128*128-pixel, 256*256-pixel, and 512*512-pixel inputs, respectively. Finally, the combination of 256*256-pixel image size and a batch size of 20 is chosen for the final training process according to the results.

The datasets used in training and testing come from two sources: 1) the original data are obtained from multiple Z-axis layers with an out-of-focus and sharp focus for each field of view. 2) Additional data were generated with a Gaussian blur from the original high-definition image to simulate the out-of-focus effect. In this way, more samples can be generated. Finally, a pair of blurry and high-resolution images of the same field of view are input into the model for training.

To ensure a stable and efficient training process and make the generator and discriminator converge, a multistep training strategy is adopted. 1) First, the generator is trained so that it can output primary-quality images. 2) Then, the discriminator is trained to identify fake images generated by the generator. 3) Finally, fire up the game and start the game process between the generator and the discriminator.

Initially, approximately 4,200 brightfield live-cell images (256*256 pixels) of the iPSC-CM differentiation process were used in the model test. The CIEGAN model only takes a few hours to achieve decent results on an NVIDIA T4. The time spent on training varies depending on the size of the training set in other applications. However, the use of the CIEGAN model is very fast; it can run 128 images (256*256 pixels) at a time of only a few minutes on VIDIA T4. Most of this is the load time of the model checkpoints. Once the model is loaded, its prediction timeliness is comparable to real-time processing. Nevertheless, our program provides automatic segmentation and assembly to handle larger input images. Finally, the performance of the CIEGAN should be similar to the same level of the CUDA computational acceleration unit. For example, it should have approximately the same time cost as this article on an NVIDIA 1080Ti GPU (12 GB).

## Results

In this section, the CIEGAN is applied to long-term live-cell imaging of iPSC-CM differentiation. It significantly facilitates the research works by enhancing time-lapse microscopy images and carrying out the next analysis work. The CIEGAN successively enhanced the brightfield image of induced cardiomyocytes and obtained many good results. Then, the method is extended to the enhancement of fluorescence images, and the results are promising. Finally, several other similar methods are compared and public databases are used to explore the practicality and scalability of these methods.

### Brightfield Image Enhancement

**FIGURE 5:**
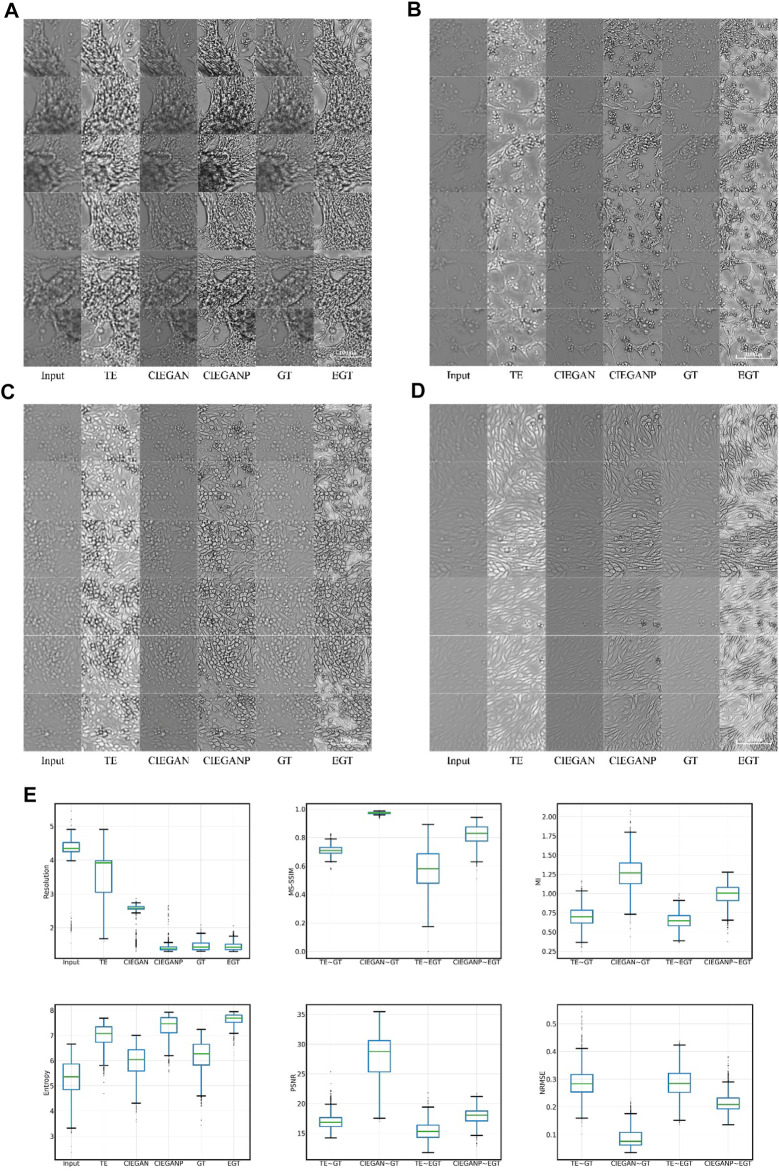
Brightfield cell image enhancement results of iPSC-CM differentiation experiments. The results for different cell morphologies are shown in subfigures **(A)**, **(B)**, **(C)**, and **(D)**. TE stands for the traditional enhancement method, CIEGAN, CIEGAN plus (CIEGANP) is our method, GT is the ground truth, and EGT is the enhanced ground truth. **(E)** Boxplot comparison results of resolution, MS-SSIM, mutual information (MI) entropy, PSNR, and NRMSE (*n* = 1128).

First, the brightfield images in the hiPSC-CM differentiation process are enhanced for qualitative testing and the results are shown in Figure 5. A variety of cell morphologies are selected to test the robustness of this method. The thickness of the observed cells varies from flat monolayer to three-dimensional structures.

In the results, the traditional enhancement method (TE) is used to enhance the blurred input images for comparison. The TE method adopts the combination of the unsharp masking (Polesel et al., 2000; Deng, 2010) and contrast limited adaptive histogram equalization (CLAHE) (Pisano et al., 1998; Reza, 2004). Unsharp masking and CLAHE are classical tools for sharpness enhancement that can adaptively adjust and enhance the sharpness and contrast of the image, respectively. Here, the TE columns in Figure 5 show the results of the enhancement of the input cell images. Although the brightness and contrast of the images have been significantly improved through the enhancement of the traditional method, the blurring problem of the image has not been fundamentally solved. However, our CIEGAN method outperforms the traditional methods and benefit from the adversarial process and perceptual loss. The results of the CIEGAN are very close to the ground truth (GT), especially on the reconstruction of the fine structure of cells.

On the other hand, inspired by the Real-ESRGAN (Real Enhanced Super-Resolution Generative Adversarial Network) (Wang et al., 2021b), an improved CIEGAN was trained by using an enhanced version of the training sets, which we call CIEGAN plus (CIEGANP). The difference between them is the training inputs; the CIEGANP model is trained with enhanced ground truth images. Here, enhanced ground truth (EGT) is the result of image enhancement using unsharp masking and CLAHE methods for the GT. Interestingly, the image enhanced by the EGT method highlights the dead cells in the image (black dots) because the size of the dead cells or impurities is much smaller than the cells. Figure 5 (A–D) shows that the result of the CIEGANP is better than that of the CIEGAN in brightness and sharpness. Moreover, the results of the CIEGANP have less pepper noise or spikey artifacts than the enhanced ground truth (EGT) due to the introduced variation regulator.

To further evaluate the method, we performed a package of quantitative evaluations between traditional methods. The normalized root mean square error (NRMSE), peak signal-to-noise ratio (PSNR), and multi-scale structural similarity index (MS-SSIM) (Wang et al., 2003) are used in the image similarity assessment between the generated image and ground truth. The NRMSE reflects the pixel difference between the two images, and the smaller the value is, the better. The PSNR is the ratio of the maximum possible power of a signal to the power of corrupting noise that affects representation fidelity, which can objectively measure the image quality; the larger the value is, the better. The MS-SSIM is an improved version of the SSIM that is also used to measure image quality; the closer the value is to 1, the better. In addition, mutual information (MI) is used to measure the similarity of two images. The mutual information I between two pictures is formulated as Eq. 12:

$$I\left(X;Y\right)=\sum_{y\in Y}\sum_{x\in X}p\left(x,y\right)\log\left(\frac{p\left(x,y\right)}{p\left(x\right)p\left(y\right)}\right).$$ (12)

Mutual information in Eq. 12 describes the reciprocity between objects in two images, and the larger the value is, the higher the similarity between the two images will be.

We also used some no-reference methods for a single image quality evaluation in addition to the full reference method. The information gain and the estimation of the resolution ratio are used for evaluation after image enhancement. The information entropy H can be expressed as Eq. 13:

$$H\left(x\right)=E\left(I\left(x\right)\right)=-\sum_{x\in X}p\left(x\right)\log\left(p\left(x\right)\right).$$ (13)

In Eq. 13, *x* represents the gray value of a pixel in the image, and p(x) is the probability of occurrence of its pixel gray value. For a normal 8-bit depth grayscale image, *X* contains the grayscale from 0–255 in this image. The information entropy is a nonnegative value, that is, it is used to describe the uncertainty of the pixels in the picture. The larger the information entropy is, the greater will be the amount of information contained in the picture.

On the other hand, resolution ratio estimation is widely used in biological image evaluation because it can indicate the actual resolution per pixel (Qiao et al., 2021). The resolution ratio calculation is performed by a decorrelation analysis, where the cross-correlation coefficient is expressed as Eq. 14 (Descloux et al., 2019):

$$d\left(r\right)=\frac{\int Re\left\{I\left(k\right)I_{n}\left(k\right)M\left(k,r\right)\right\}dk_{x}dk_{y}}{\sqrt{\int {\left|I\left(k\right)\right|}^{2}dk_{x}dk_{y}\int {\left|I_{n}\left(k\right)M\left(k,r\right)\right|}^{2}dk_{x}dk_{y}}}.$$ (14)

In Eq. 14, *k* is the Fourier space coordinates, and *I* is the Fourier transform function. I(k) represents the Fourier transform of the input image, and $I_{n}\left(k\right)$ represents the normalization of I(k) . M(k,r) is the circular mask of the radius r (Descloux et al., 2019). The input image is passed through a series of high-pass filters and found the local maximum of the highest frequency; the normalized frequencies were denoted as $k_{c}$ . The resolution ratio is $resolution=\frac{2p}{k_{c}}$ , where *p* is the pixel size in photo acquisition.

The resolution ratio can measure the recognizability of structures in biological images; the smaller the resolution ratio value is, the greater the accuracy will be. The physical resolution in the iPSC-CM experiments is 0.65 μM per pixel, while the actual resolution of the ground truth images obtained may be poorer: 1.465 μM on average. The input images have a resolution of 4.305 μM on average due to out-of-focus, and our method can enhance this to 2.488 μM by the CIEGAN on average and 1.416 μM by the CIEGANP on average. Here, the traditional enhancement (TE) method only has a resolution of 3.546 μM on average. The results of the boxplot comparison are shown in Figure 5E.

An expert questionnaire is conducted to investigate whether the resulting pictures generated by this model are suitable for scientific research purposes. The results also show that the CIEGANP generally performs better than the other methods and has appropriate contrast and brightness.

### DAPI Image Enhancement

**FIGURE 6:**
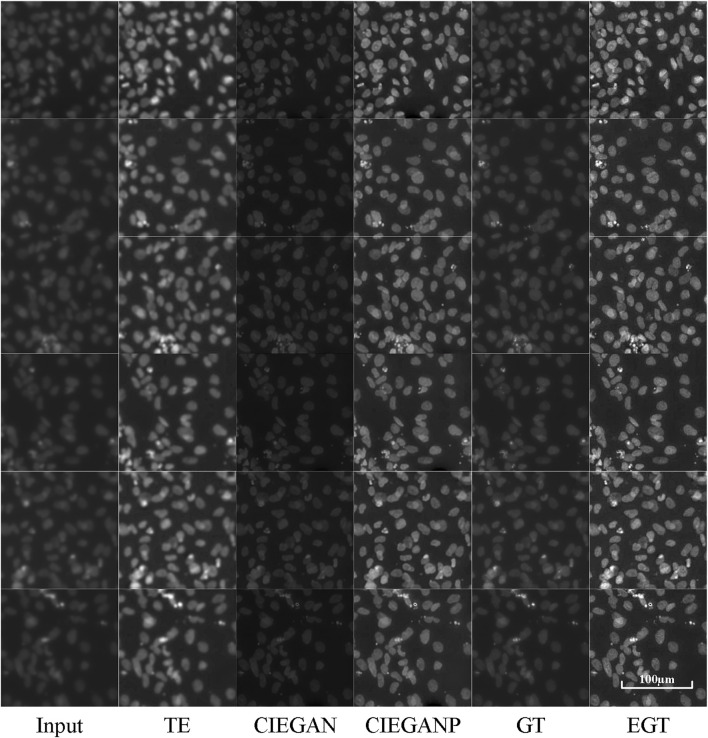
Enhancement results of Hoechst 33342 fluorescence microscopy images of the iPSC-CM differentiation experiment. TE stands for the traditional enhancement method, CIEGAN and CIEGAN plus (CIEGANP) are our methods, GT is the ground truth, and EGT is the enhanced ground truth.

The assessment of cardiomyocyte quality is required after the third stage of differentiation (Figure 3) in the iPSC-CM differentiation process. Therefore, fluorescent staining experiments were performed. Here, we stained two types of cell markers: cardiomyocyte-specific cTnT antibody and the nucleus-specific Hoechst 33342, which are used to assess the differentiation ratio and the cardiomyocyte quality. The enhancement results of the cell image stained with Hoechst 33342 are shown in Figure 6.

The CIEGAN algorithm has significantly enhanced the sharpness and contrast of the blurred cell staining images. It even obtained a higher signal-to-noise ratio than the ground truth. However, the performance in brightness is not perfect, which is a common problem of this model. The reason for this is that there are many black images with nothing in the training set. For this reason, the model trained with sharped ground-truth images as the CIEGAN plus has been introduced, and it significantly improves the sharpness and brightness of generating biological images while increasing the signal-to-noise ratio.

### CTnT Image Enhancement

**FIGURE 7:**
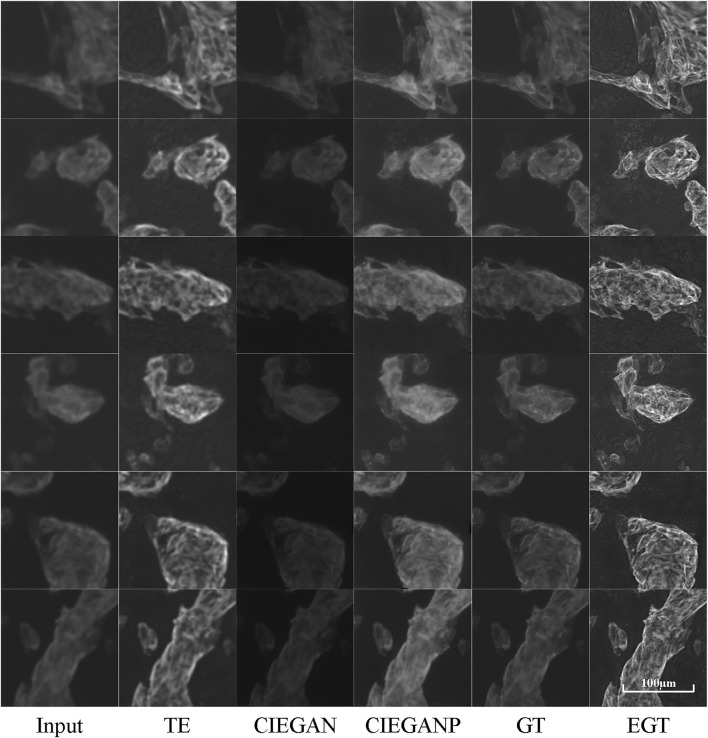
Results of cTnT fluorescence microscopy images of the iPSC-CM differentiation experiment. TE stands for the traditional enhancement method, CIEGAN, CIEGAN plus (CIEGANP) is our method, GT is the ground truth, and EGT is the enhanced ground truth.

The enhanced cTnT fluorescent stained images are shown in Figure 7. The CIEGAN model achieves excellent generalization performance for different types of fluorescent images. The phototoxicity can be ignored in this experiment because the fluorescent staining method kills cells. Longer and stronger exposures can be used for imaging. However, photobleaching cannot be ignored because of the poor stability of dyes under strong light irradiation. It is not possible to use a strong intensity of light for a long time during the exposure process. Therefore, the balance between exposure time, light intensity, and image clarity also needs to be considered when taking photographs of fluorescence microscopy images. Nevertheless, it is sometimes impossible to obtain fluorescent photographs again because of severe photobleaching. In this case, the CIEGANP can not only deal with various out-of-focus images but can also enhance images with a low signal-to-noise ratio due to photobleaching.

### Comparison With Other Methods

**FIGURE 8:**
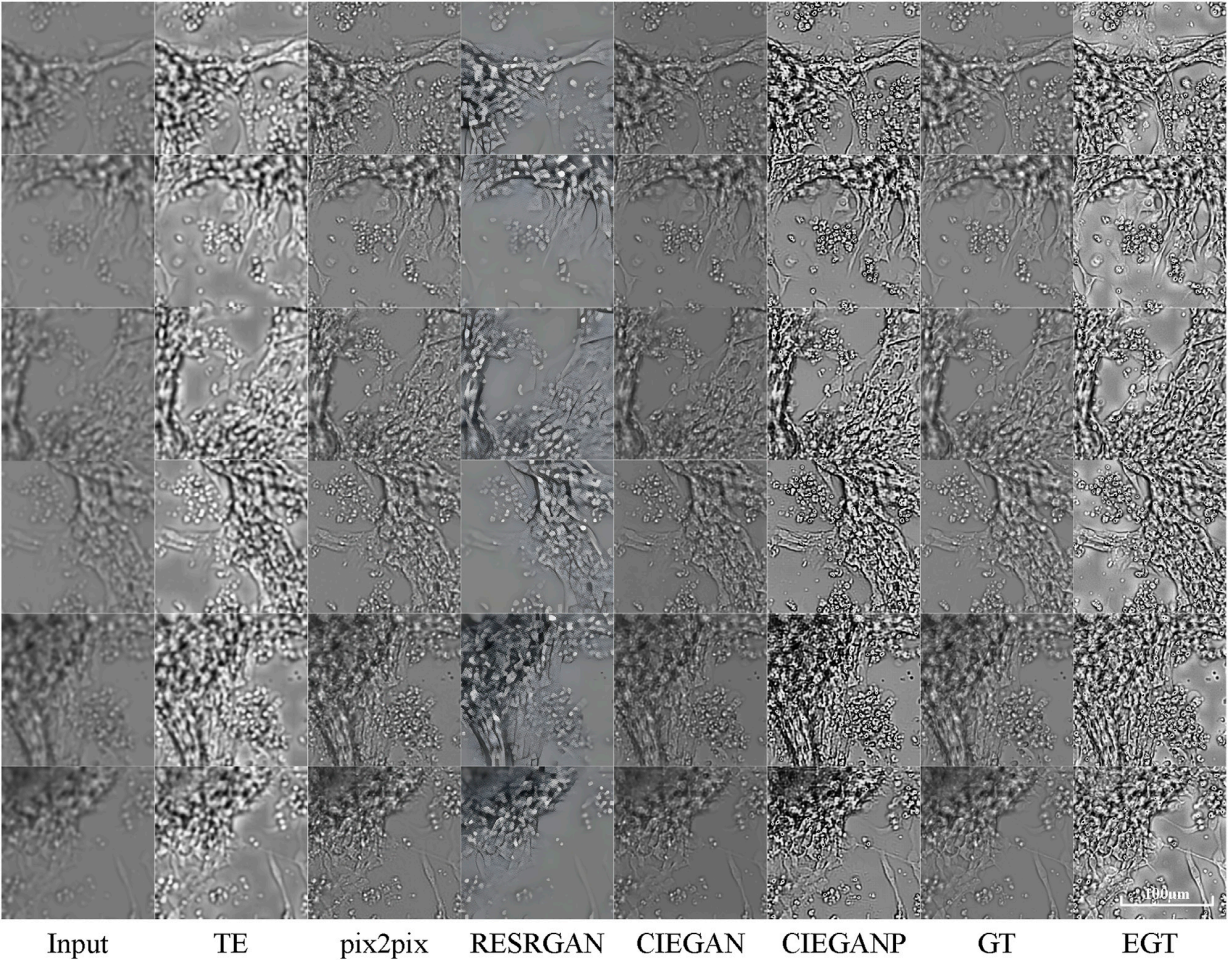
Comparison results of brightfield image enhancement of the iPSC-CM differentiation experiment. TE stands for the traditional enhancement method, CIEGAN, CIEGAN plus (CIEGANP) is our method, GT is the ground truth, and EGT is the enhanced ground truth. “pix2pix” is the method proposed by Isola et al. (2017) and RESRGAN is the real-ESRGAN method proposed by Wang et al. (2021b).

The comparison with other methods is also carried out here. The “pix2pix” proposed by Isola et al. (2017) and the Real-ESRGAN proposed by Wang et al. (2021b) are used for comparison. The comparison results are shown in Figure 8.

First, the “pix2pix” model is trained on the dataset of brightfield images in the hiPSC-CM experiments and then achieves convergence. The results of “pix2pix” show its excellent performance, but there are a small number of artifacts and compression blur. On the other hand, the Real-ESRGAN shows its remarkable performance in real-world photos. Here, the Real-ESRGAN can increase the input biological image from 256*256 pixels to 1,024*1,024 pixels, which is 16 times the quantity of pixels. Because it was designed to perform super-resolution enhancement instead of dealing with blur degradations in biological micrographs, the high-definition pictures generated by the Real-ESRGAN are biologically distorted.

## Discussion

To overcome challenges in long-term live-cell imaging, this study proposes a cell image-enhanced generative adversarial network (CIEGAN). This method can resolve various blurred degradations in biological brightfield cell images and significantly improve the image space resolution ratio. It can maximize the effectiveness of the information mining of the biological image. Needless to say, it is very convenient to make the blurred images sharp again with a few steps. Moreover, it accelerated the imaging speed because there is no need to take multiple Z-axis layers to prevent out-of-focus problems. It creates more time for experimental throughput, so researchers can investigate more conditions or increase the frequency of image acquisition. Most importantly, many cellular dynamic response processes are rare and quick and do not give us a second chance to recapture the study of cell differentiation and reprogramming. Here, the CIEGAN can give researchers a second chance to reproduce sharp biological images in a short time. Furthermore, it can handle imaging mishaps during cell culture: cell clumping bulging, blurring caused by floating dead cells or the poor clarity of the medium, out-of-focus problems caused by thermal expansion and contraction of the culture chamber, and even the blur caused by the beating of differentiated mature cardiomyocytes, etc. Nevertheless, the image enhancement process is fast, of low cost, and can easily be extended to other applications. It is convenient for researchers to reproduce the developmental trajectories of cell lines from long-term time-lapse unstable image streams.

On the other hand, the blurred cTnT staining results of myocardial cells could be enhanced by the aforementioned method. It is necessary to photograph Z-stacks to ensure full-field vision, as monolayer cardiomyocytes are still stereoscopic (Christiansen et al., 2018). The CIEGAN can obtain clear cTnT staining images from single-layer imaging and reduce the requirements and complexity of microscopic photography. In addition, sharp images have more cell features, such as the sarcomere structure of cardiomyocytes, which can indicate the state of maturity of the cardiomyocytes (Veerman et al., 2015). This method can not only be applied to cardiomyocytes but also to enhance the image of other cells, such as neurons, hepatocytes, adipocytes, etc., which will obtain more valuable information for biological-image study for further application.

Notably, once the deep learning model was trained, the model performed well on the same cell type of microscopic images. It is best to retrain the model to generalize other types of cells. The performance of the model is positively correlated with the sharpness of the input training examples. Therefore, researchers cannot expect this model to perform well on poor training data sets. This model also has some common limitations similar to other deep learning models. Because image transformation with deep learning models is not perfect in any way, real-world situations tend to be more complex (Cai et al., 2019; Yang et al., 2019; Qiao et al., 2021). The deep learning model cannot predict new or unseen fine structures limited by the image morphology and granularity of the training set, which is also a great challenge faced by the industry. Therefore, improving the quality of the first-hand images obtained by the microscope is a fundamental and indispensable part of biological studies. On the other hand, the method proposed in this study can improve the image quality in long-term living cell images to its best. It is very helpful in saving time, especially in long-term live-cell imaging with long experimental periods. Because it is impossible to repeat photograph processing due to the rare phenomenon of photobleaching, another time-consuming biological experiment must be restarted.

In further research, the CIEGAN will be improved by introducing more advanced generator structures or more penalty functions. U-net is becoming widely used in deep learning processing schemes for biological image processing (Ounkomol et al., 2018; Weigert et al., 2018; Kandel et al., 2020; Dance, 2021; Wieslander et al., 2021). It is widely implemented in image segmentation and classification thanks to its structure of directly copying the feature maps of convolutional layers to deconvolutional layers (Ronneberger et al., 2015). We can try to introduce this network mechanism into our model and in addition, the concept of the network structure of GoogLeNet (Szegedy et al., 2015, 2016, 2017). The method in this study mainly uses multiple image difference losses as the training criteria for the GAN generator, and more losses could be tried in the next step.

The CIEGAN method has high scalability and broad application prospects in image enhancement scenarios, which can help biologists observe and investigate image phenomena in the process of cell differentiation and reprogramming more intuitively and deeply. In turn, more efficient experimental models can be designed, and even effective potential treatments for related diseases can be found. We will continue to refine the application of the CIEGAN method to more image enhancement scenarios.

## Data Availability

The original contributions presented in the study are included in the article/Supplementary Material; further inquiries can be directed to the corresponding authors.
