# Supplementary material for: CIEGAN: A Deep Learning Tool for Cell Image Enhancement
Source: Front Genet. 2022 Jul 4;13:913372. doi: 10.3389/fgene.2022.913372 (PMC9298179; doi:10.3389/fgene.2022.913372)
Supplement: Supplementary file 1 [file DataSheet2.PDF]

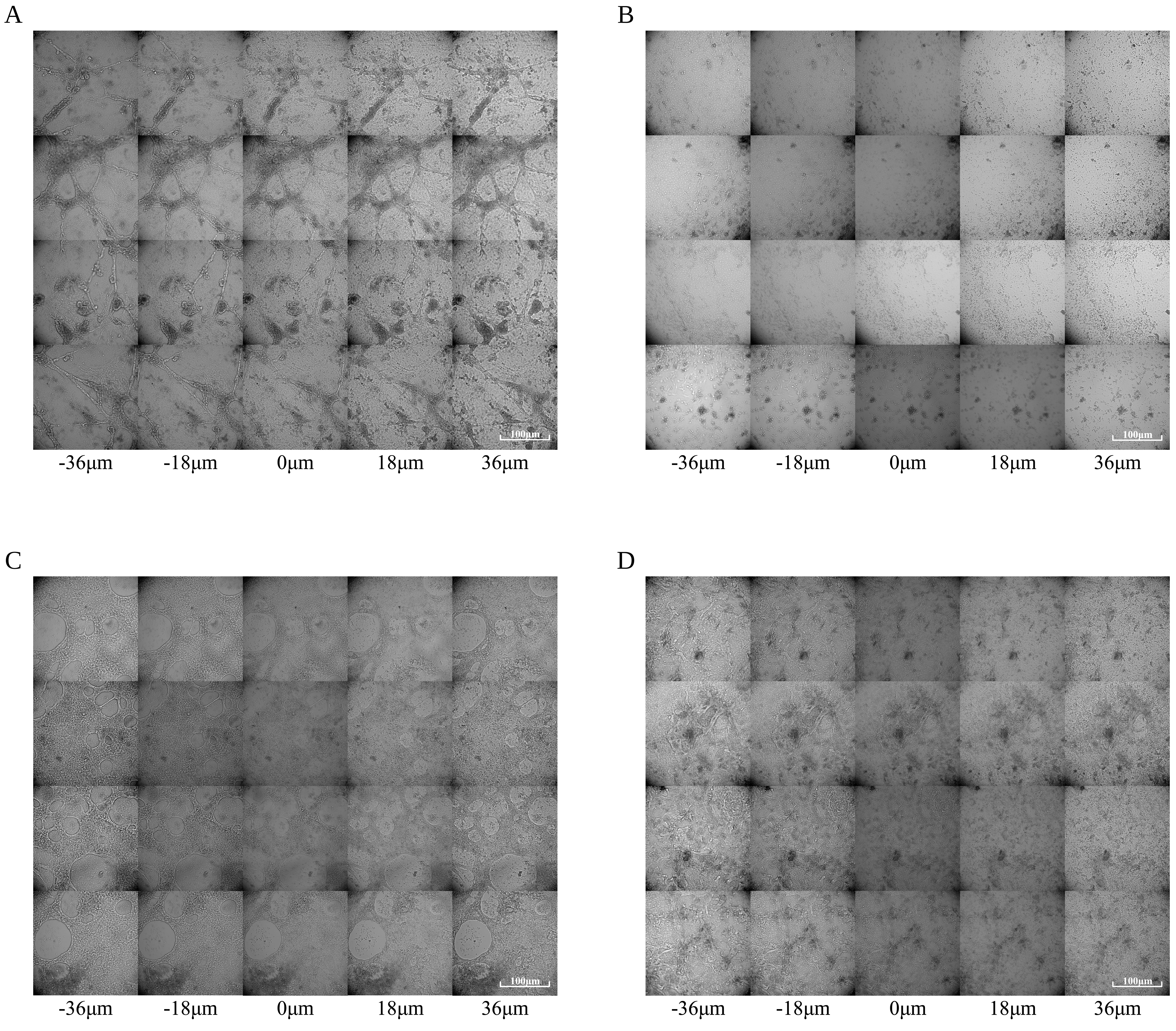

Supplementary Figure 2. Different Z-layer images of the same field of the iPSC-CM differentiation experiment. The results for different cell morphologies are shown in subfigures (A), (B), (C) and (D). Five layers at 18 μm intervals with total vertical distances of 72 μm (from -36 μm to 36 μm) were obtained for the study. Zero microns is the intermediate image layer with the sharpest focus. Different Z layers can reveal different information for cells with a three-dimensional structure and zoom in for the best view. There is no optimal focal layer for the chaotic views, and the observation cannot attend to one thing without neglecting the other in one image.
