# Supplementary material for: CIEGAN: A Deep Learning Tool for Cell Image Enhancement
Source: Front Genet. 2022 Jul 4;13:913372. doi: 10.3389/fgene.2022.913372 (PMC9298179; doi:10.3389/fgene.2022.913372)
Supplement: Supplementary file 2 [file DataSheet1.PDF]

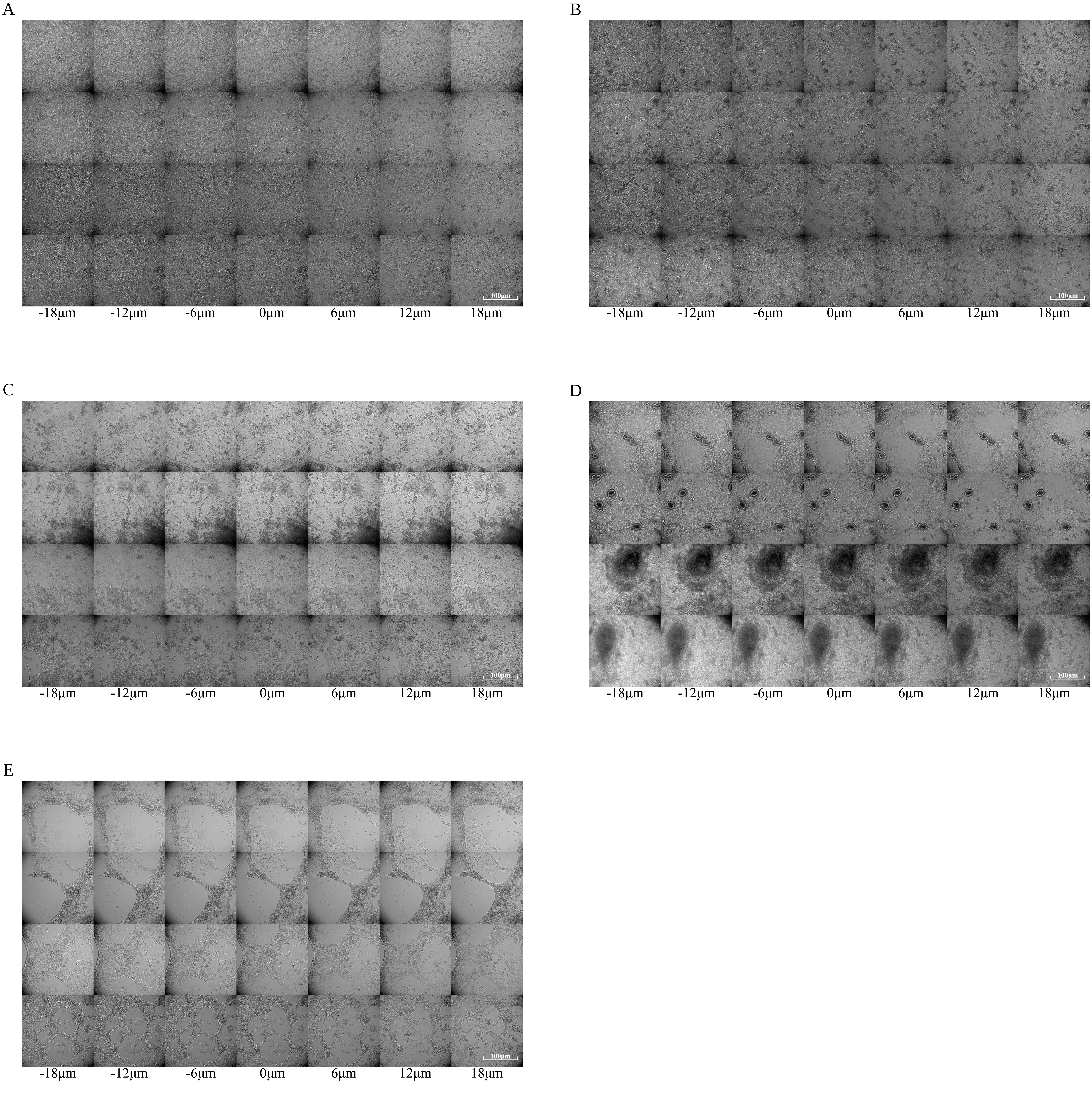

Supplementary Figure 1. Different Z-layer images of the same field of the iPSC-CM differentiation experiment. The results for different cell morphologies are shown in subfigures (A), (B), (C), (D) and (E). Seven layers at 6 µm intervals with total vertical distances of 36 µm (from -18 µm to 18 µm) were obtained for the study. Zero microns is the intermediate image layer with the sharpest focus. Different Z layers can reveal different information for cells with a three-dimensional structure and zoom in for the best view.
